# Hypertension care: the knowledge and attitudes of the community pharmacists

**DOI:** 10.1080/20523211.2025.2544635

**Published:** 2025-09-05

**Authors:** Aliki Peletidi, Ioannis Doundoulakis, Eleni Vavoulioti, Christos Petrou, Michael Petrides

**Affiliations:** a Department of Health Sciences, School of Life and Health Sciences, University of Nicosia, Nicosia, Cyprus; b Bioactive Molecules Research Center, School of Life and Health Sciences, University of Nicosia, Nicosia, Cyprus; c Faculty of Science Engineering and Computing, Kingston University, Kingston upon Thames, UK; d Heart Rhythm Management Centre, European Reference Networks Guard-Heart, Postgraduate Program in Cardiac Electrophysiology and Pacing, Universitair Ziekenhuis Brussel Heart Rhythm Research Brussels, Vrije Universiteit Brussel, Brussels, Belgium

**Keywords:** Hypertension screening, hypertension management, community pharmacists, primary healthcare, Greece

## Abstract

**Background:**

Hypertension is a major global risk factor for cardiovascular disease and mortality. In Greece, prevalence is about 40%, with many cases undiagnosed or poorly managed. While doctors remain central to diagnosis and treatment, community pharmacists, as accessible healthcare professionals, can support early detection and ongoing management. This study assesses Greek community pharmacists' knowledge of hypertension detection and management, focusing on their ability to measure blood pressure accurately, categorise hypertension, and understand lifestyle factors.

**Methods:**

A cross-sectional survey was conducted with 92 community pharmacists in Greece, using a structured questionnaire to assess knowledge of blood pressure measurement, hypertension categorisation, and lifestyle influences. The instrument collected demographic data and responses on hypertension management practices. Data were gathered between December 2022 and April 2023 and analysed using SPSS v22.

**Results:**

The survey revealed moderate to good knowledge levels among pharmacists, particularly in blood pressure measurement (mean score: 71.74%, SD = 16.08) and hypertension categorisation (mean score: 76.9%, SD = 8.48). Knowledge about lifestyle impacts on blood pressure was lower (mean score: 53.57%, SD = 15.65). Younger pharmacists (26 36 years old) demonstrated significantly higher lifestyle impact knowledge (p = 0.013) and overall knowledge scores (p = 0.012) compared to the rest of the age groups, whereas pharmacists with postgraduate degrees had significantly higher scores in blood pressure measurement (p = 0.009) and overall knowledge scores (p = 0.002) compared to those with only a tertiary education.

**Conclusion:**

Findings underscore early undergraduate training and continuous professional development to strengthen pharmacists' hypertension-management role. Targeted programmes should deepen understanding of lifestyle determinants. Greek community pharmacists are well positioned for early detection within multidisciplinary care, yet more education is needed to optimise impact. The Panhellenic Pharmaceutical Association and Federation of Cooperative Pharmacists of Greece advocate recognising pharmacists' role in structured, advanced services, including hypertension management, to improve public health in Greece.

## Background

Hypertension is a major global public health challenge and one of the leading preventable causes of cardiovascular disease, stroke, and premature death (Giles et al., 2009) (World Health Organization, 2023). Its diagnostic thresholds may vary depending on comorbidities; for example, in diabetic patients, hypertension may be defined as blood pressure exceeding 130/80 mmHg, while for the general population, hypertension is typically diagnosed when systolic blood pressure is ≥140 mmHg and/or diastolic blood pressure is ≥90 mmHg (Williams et al., 2018). If left untreated, elevated blood pressure can result in serious complications, including myocardial infarction, stroke, heart failure, peripheral vascular disease, atherosclerosis, atrial fibrillation, and chronic kidney disease (Slivnick & Lampert, 2019; Song et al., 2020). It is a leading cause of cardiovascular diseases and contributes significantly to morbidity and mortality worldwide. The condition is associated with a range of risk factors, including age, gender, lifestyle, and genetic predisposition (Jeemon et al., 2021; Wijaya et al., 2020). Lifestyle factors such as unhealthy diet, physical inactivity, excessive alcohol consumption, and smoking are known to exacerbate hypertension (Silva et al., 2022; Del Punta et al., 2022; Stergiou et al., 2020).

Globally, hypertension affects approximately 30–45% of the adult population, and its prevalence increases with age, particularly among adults over 40 years of age (Mills et al., 2020; Williams et al., 2018). In Greece, recent national data report that hypertension affects approximately 40% of the adult population, with higher prevalence observed among men (45.4%) compared to women (35.3%) (Stergiou et al., 2021; Touloumi et al., 2020). Among hypertensive individuals in Greece, only 54% have their blood pressure adequately controlled, while approximately one-third remain undiagnosed or untreated (Di Palo & Kish, 2018; Stergiou et al., 2021). This substantial burden of undiagnosed and uncontrolled hypertension underlines the urgent need for improved screening, detection, and management strategies.

Effective management of hypertension requires early detection, patient education, lifestyle modifications, medication adherence, and continuous monitoring. Community pharmacists are uniquely positioned to contribute to these aspects of care due to their accessibility, frequent patient interactions, and expanding role within primary healthcare systems. As highly accessible healthcare professionals, community pharmacists often serve as the first point of contact for individuals seeking medical advice or treatment for minor ailments. Their frequent interactions with patients allow pharmacists to play a pivotal role in the early detection and ongoing management of hypertension. They routinely provide blood pressure monitoring services, lifestyle counselling, medication management, and patient education. Pharmacies are increasingly equipped with validated sphygmomanometers, enabling pharmacists to conduct accurate blood pressure measurements and identify individuals who may be unaware of their hypertensive status (Ahmed et al., 2011; Roy et al., 2020).

International evidence consistently highlights the positive impact of pharmacist-led interventions on hypertension outcomes. Numerous randomised controlled trials and meta-analyses have demonstrated that pharmacist involvement can lead to significant improvements in blood pressure control, medication adherence, and cardiovascular risk reduction. For instance, pharmacist-led interventions have been associated with reductions in systolic blood pressure ranging from 7 to 10 mmHg and improved hypertension control rates across various healthcare settings (Al Hamarneh et al., 2013; De Ávila Machado et al., 2017; Mills et al., 2020; Santschi et al., 2014).

Pharmacists contribute to hypertension care by conducting blood pressure measurements, providing guideline-based counselling, identifying drug-related problems, supporting therapeutic adjustments in collaboration with physicians, and promoting patient self-monitoring and lifestyle changes, such as dietary modifications, physical activity, and smoking cessation (Cheema et al., 2018; Santschi et al., 2015; Shoji et al., 2019). Their role as medication experts allows them to optimise pharmacotherapy, address adherence barriers, and ensure patients understand their treatment plans (Reeves et al., 2021).

## The Greek context

In Greece, community pharmacies are widespread and serve as an integral part of the healthcare system, often acting as the first healthcare contact for many patients. Pharmacists in Greece increasingly engage in chronic disease management, including hypertension monitoring and counselling. However, despite their growing involvement, there remains limited empirical data evaluating Greek pharmacists’ knowledge, preparedness, and training needs specific to hypertension management.

Given the high prevalence of hypertension in the Greek population and the evolving role of pharmacists in chronic disease care, understanding their current knowledge base is crucial for informing targeted educational programmes, shaping healthcare policy, and ultimately improving patient outcomes. While international studies have identified knowledge gaps among pharmacists in areas such as hypertension guidelines, patient counselling, lifestyle modification, and drug interaction management, it remains unclear whether similar gaps exist among Greek community pharmacists.

To address this gap, the present study aimed to assess the knowledge of community pharmacists regarding hypertension detection, monitoring, and management. The study sought to identify specific knowledge gaps and highlight opportunities for targeted education and continuing professional development. By providing data from Greece – a healthcare context underrepresented in existing literature – this research contributes valuable insights into the preparedness of pharmacists to assume a more prominent role in hypertension care.

## Methods

### Type of study – sampling method

This study employed a quantitative cross-sectional design to evaluate the knowledge of community pharmacists in Greece regarding hypertension management. A cross-sectional design was chosen to provide a snapshot of the current knowledge levels and practices among pharmacists at a specific point in time. No formal statistical sample size calculation was conducted, as the study was exploratory in nature. A convenience sampling method was used, as no comprehensive national registry of pharmacists was publicly available. We recruited participants from the membership list of the Federation of the Cooperative Pharmacists of Greece (OSFE), focusing on community pharmacists practicing in the Piraeus area (Attica Region). Pharmacists were invited to participate through OSFE member communications, direct email invitations, and in-person visits at selected pharmacies. Those who voluntarily agreed to participate were included in the study. The sample aimed to include pharmacists from a range of ages, genders, and years of experience to reflect the diversity of the local pharmacist population. Inclusion criteria were: licensed community pharmacists (either employees or owners) actively practicing in the Piraeus region, members of OSFE, and willing to provide informed consent. Exclusion criteria included retired pharmacists, pharmacy assistants, hospital pharmacists, or pharmacists practicing outside the Piraeus region.

### Questionnaire development, validity, and reliability

The questionnaire was developed based on an extensive review of the existing literature on pharmacists’ knowledge of hypertension management and relevant international guidelines (Williams et al., 2018). The literature review was performed independently by the authors: [AP, PhD, MPharm, Assistant Professor in Clinical Pharmacy Practice] and [MP, BPharm, MSc in Pharmacy, PhD (c) in Pharmacoepidemiology].

To establish content validity, a panel of five experts – including clinical pharmacists, academic researchers, and practicing community pharmacists – reviewed the questionnaire for relevance, clarity, and comprehensiveness. Face validity was assessed through cognitive interviews with three practicing pharmacists to ensure that the questions were understandable and interpreted as intended. A pilot study was conducted with 10 pharmacists who were not part of the final sample to assess feasibility and refine the instrument based on participant feedback. Internal consistency reliability was evaluated using Cronbach’s alpha coefficient, which yielded an acceptable value of 0.81, indicating good reliability of the scale (Taber, 2017). The final questionnaire comprised both closed-ended questions and Likert-scale items assessing pharmacists’ knowledge in five key domains: blood pressure measurement techniques, hypertension categorisation, home blood pressure monitoring, lifestyle modifications, and demographic data.

The questionnaire consisted of four sections: (1) demographic characteristics; (2) blood pressure measurement techniques; (3) knowledge on hypertension classification and guidelines; and (4) lifestyle and management recommendations. It included multiple-choice, single-response, and Likert-scale questions. The questionnaire contained a total of 48 items.

### Knowledge score calculation

Each knowledge item was scored as correct or incorrect. The total knowledge score was calculated by summing the number of correct responses and converting the score to a percentage (%). The final overall knowledge score ranged from 0% to 100%.

### Data collection procedure

The data collection process took place from December 2022 to April 2023. To maximise participation, the questionnaire was distributed to community pharmacists across Piraeus (Attica Region, Greece) using a combination of electronic methods (via the Unic Moodle platform) and face-to-face distribution at pharmacies. In both formats, the questionnaire was self-administered. During face-to-face distribution, trained research assistants delivered the paper-based questionnaires but did not provide explanations or read the questions aloud to avoid interviewer bias. Participation was voluntary, and anonymity was maintained throughout the process to encourage honest responses.

### Ethical considerations

Ethical considerations were meticulously observed throughout the study. All participants received clear and prior information about the study's objectives and the confidentiality and anonymity of their responses, which were exclusively used for research purposes. Informed consent was given upon agreement in participating of the study. The voluntary nature of the study was emphasised, with the understanding that completing the questionnaire implied implicit consent to participate. Ethical approval was taken prior to data collection from the Federation of the Cooperative Pharmacists of Greece (OSFE).

### Statistical analysis

The analysis of the collected data was carried out using SPSS v22. Descriptive statistics were employed to summarise the demographic characteristics of the participants and their knowledge scores across different areas. To identify significant differences in knowledge scores based on demographic variables such as age, gender, education level, and years of experience, inferential statistics were applied, including non-parametric tests like the Mann Whitney U test and Kruskal Wallis H test, as well as Spearman correlation, due to the non-normal distribution of the data. A *p*-value of less than 0.05 was regarded as statistically significant, ensuring a high level of confidence in the findings. Although multivariate regression was considered, it was not conducted due to the modest sample size.

## Results

1. Demographics and Descriptive Statistical Analysis

**Figure 1.**
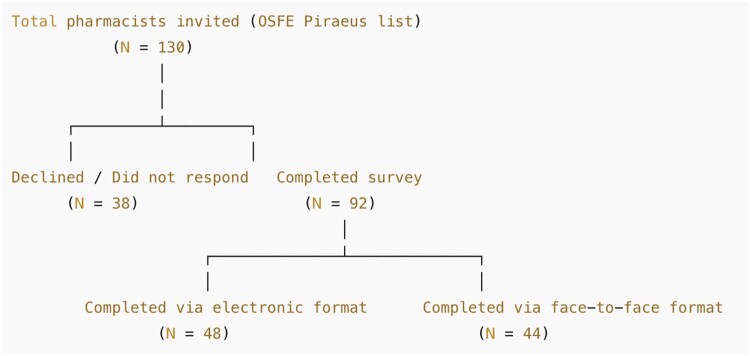
Participant recruitment and data collection flow diagram.

A total of 130 community pharmacists in Piraeus were invited to participate in the study. Of these, 92 pharmacists completed the survey, resulting in a participation rate of 70.8%. Among the respondents, 48 pharmacists completed the questionnaire electronically via the Unic Moodle platform, while 44 pharmacists participated through face-to-face paper-based data collection (Figure 1). On average, participants required approximately 20 minutes (range: 15–30 minutes) to complete the questionnaire.

The sample was composed of 45 male pharmacists (48.9%, n = 45) and 47 female pharmacists (51.1%, n = 47). The distribution of participants across age groups showed that most pharmacists were aged between 37–47 years (38.0%, n = 35), followed by those aged 48–58 years (34.8%, n = 32). Pharmacists aged 26–36 years represented 17.4% of the sample (n = 16), while 9 pharmacists (9.8%, n = 9) were older than 58 years (Table 1).

**Table 1.** Demographic characteristics of the sample.

		Frequency	Percentage (%)
Gender	Male	45	48.9
	Female	47	51.1
Age	26–36	16	17.4
	37–47	35	38.0
	48–58	32	34.8
	>58	9	9.8
Education	Tertiary (MPharm/BPharm)	58	63.0
	Postgraduate	34	37.0
Experience (in years)	<5	9	9.8
	5–15	24	26.1
	16–25	43	46.7
	26–35	11	12.0
	36–45	5	5.4
Pharmacy ownership status	Owners	90	97.8
	Employees	2	2.2

In terms of educational background, 58 pharmacists (63.0%, n = 58) had completed a tertiary education degree, which in Greece corresponds to Bpharm (currently as MPharm) earned after a five-year university programme. The remaining 34 pharmacists (37.0%, n = 34) had pursued additional postgraduate education, including MSc or PhD qualifications. Regarding years of experience, 43 pharmacists (46.7%, n = 43) had between 16–25 years of professional experience. Pharmacists with 5–15 years of experience accounted for 26.1% (n = 24), while those with less than 5 years of experience represented 9.8% (n = 9). Pharmacists with 26–35 years of experience comprised 12.0% (n = 11), and those with 36–45 years of experience constituted 5.4% (n = 5). Most of the participants were pharmacy owners (97.8%, n = 90), while only 2.2% (n = 2) were employed as pharmacy staff. All participants practiced within the municipality of Piraeus.

With regard to blood pressure measurement equipment used in pharmacies, Microlife® devices were most common, reported by 42.4% (n = 39) of respondents, followed by Omron® devices at 39.1% (n = 36). Less frequently reported brands included Kessler® (8.7%, n = 8), Focal® (2.2%, n = 2), Nissei® (2.2%, n = 2), Riester® (1.1%, n = 1), And® (1.1%, n = 1), and Alpk2® (1.1%, n = 1). When specifying device type, the M3 sphygmomanometer was most frequently mentioned (16.3%, n = 15), followed by analogue sphygmomanometers (7.6%, n = 7), electronic arm metres (6.5%, n = 6), and other models such as the M6, M2, mercury sphygmomanometer, electric wrist metres, and Rossmax arm metres (Table 2).

**Table 2.** Blood pressure measurement by brand and type.

Brand/Type	Frequency (N)	Percent (%)
Brand of Machine: Microlife®	39	42.4
Focal®	2	2.2
Omron®	36	39.1
Kessler®	8	8.7
Nissei®	2	2.2
Riester®	1	1.1
And®	1	1.1
Alpk2®	1	1.1
Type of Meter: Arm Sphygmomanometer	3	3.3
Analogue Sphygmomanometer	7	7.6
M3	15	16.3
M2	3	3.3
Mercury	1	1.1
M6	4	4.3
Electronic Arm Meter	6	6.5
Rossmax (Arm)	1	1.1
Electric Wrist Meter	2	2.2

The study meticulously evaluated the role of community pharmacists in managing hypertension, revealing various strengths and areas for enhancement in their practices and knowledge.

### Personnel involvement and roles

The majority of blood pressure measurements were performed by the pharmacists themselves (76.1%, n = 70), while pharmacist assistants performed the measurements in 17.4% (n = 16) of cases. In 4.3% (n = 4) of cases, measurements were conducted either by the pharmacist or assistant depending on availability, and in a very small number of cases, measurements were performed by patients or whoever was available (2.2%, n = 2) (Table 3, Subtable 1.1).

**Table 3.** Summary of results for community pharmacists on hypertension management.

Subtable	Description	Options	N	Percent (%)
1.1	Personnel Performing Blood Pressure Measurements in the Pharmacy	Pharmacist	70	76.1
		Pharmacist’s Assistant	16	17.4
		Patient Themselves	1	1.1
		Assistant or Pharmacist (Available)	4	4.3
		Whoever is Available	1	1.1
		Total	92	100.0
1.2	Specialised Training or Information on Hypertension Guidelines Post-Degree	Yes	73	79.3
		No	19	20.7
		Total	92	100.0
1.3	Basis of Hypertension Guideline Updates	Informed by 2018 Guidelines	36	39.1
		Informed Before 2018	56	60.9
		Total	92	100.0
1.4	Patient Posture for Accurate Blood Pressure Measurement	Without Back Support	1	1.1
		With Back Support	91	98.9
		Total	92	100.0
1.5	Cuff Placement for Blood Pressure Measurement	Centre over Pulse Point	9	9.9
		Lower Edge 2–3 cm Above	13	14.3
		Even Pressure with Finger Width	4	4.4
		All of the Above	65	71.4
		Total	91	100.0
1.6	Frequency of Blood Pressure Measurement in the Pharmacy	Once	7	7.6
		Twice	60	65.2
		Thrice	24	26.1
		Four Times	1	1.1
		Total	92	100.0
1.7	Time Interval Between Blood Pressure Measurements	1 min	2	2.3
		2 min	18	20.2
		3 min	14	15.7
		5 min	37	41.6
		10 min	14	15.7
		15 min	3	3.4
		480 min	1	1.1
		Total	89	100.0
1.8	Agreement on Measuring Both Arms for Hypertension Diagnosis	Strongly Disagree	9	9.8
		Disagree	5	5.4
		Neutral	9	9.8
		Agree	12	13.0
		Strongly Agree	57	62.0
		Total	92	100.0
1.9	Incorrect Pre-conditions for Blood Pressure Measurement	Avoid Talking	6	6.5
		Can Eat/Smoke/Exercise	79	85.9
		Should Rest	7	7.6
		Total	92	100.0
1.10	Use of Uniform Arm Cuffs for Blood Pressure Measurement	Strongly Disagree	63	68.5
		Disagree	6	6.5
		Neutral	10	10.9
		Agree	4	4.3
		Strongly Agree	9	9.8
		Total	92	100.0
1.11	Impact of Cuff Size on Blood Pressure Accuracy	Strongly Disagree	3	3.3
		Neutral	3	3.3
		Agree	10	10.9
		Strongly Agree	76	82.6
		Total	92	100.0
1.12	Frequency of Sphygmomanometer Maintenance	Annually	9	9.8
		Every Two Years	51	55.4
		Every Three Years	30	32.6
		Never	2	2.2
		Total	92	100.0
1.13	Procedure for Arm Discrepancy in Blood Pressure Measurement	Higher Reading	88	95.7
		Lower Reading	4	4.3
		Total	92	100.0
2.1	Detection Levels of Normal Blood Pressure	120–129 mmHg Systolic, 80–84 mmHg Diastolic	43	46.7
		120–129 mmHg, 90–99 mmHg	2	2.2
		120–139 mmHg, 80–84 mmHg	15	16.3
		120–139 mmHg, 80–89 mmHg	32	34.8
		Total	92	100.0
2.2	Detection Levels of Stage 1 Hypertension	140–159 mmHg Systolic, 80–84 mmHg Diastolic	14	15.2
		140–159 mmHg, 90–99 mmHg	77	83.7
		140–159 mmHg, 100–109 mmHg	1	1.1
		Total	92	100.0
2.3	Detection Levels of Stage 2 Hypertension	160–179 mmHg Systolic, 100–109 mmHg Diastolic	62	67.4
		160–179 mmHg, 90–99 mmHg	26	28.3
		160–179 mmHg, 80–84 mmHg	4	4.3
		Total	92	100.0
2.4	Detection Levels of Stage 3 Hypertension	180 mmHg Systolic, > 105 mmHg Diastolic	20	21.7
		180 mmHg, > 110 mmHg	65	70.7
		185 mmHg, > 110 mmHg	7	7.6
		Total	92	100.0
3.1	Impact of Untimely Detection of Hypertension	Agree	3	3.3
		Strongly Agree	87	94.6
		Neutral	2	2.2
		Total	92	100.0
3.2	Diseases Increasing Blood Pressure	Chronic Kidney Disease	5	5.4
		Overactive Adrenal Gland	1	1.1
		All of the Above	85	92.4
		Total	92	100.0
3.3	Characteristics of White Coat Hypertension	Elevated in Doctor’s Office Only	92	100.0
3.4	Best Detection Method for Community Hypertension	Pharmacy Measurement	88	95.7
		Workplace Measurement	3	3.3
		Community Events	1	1.1
		Total	92	100.0
4.1	Frequency of Home Blood Pressure Measurement	Twice Daily (Correct)	24	26.7
		Each Morning	44	48.9
		Each Day	6	6.7
		1–3 Times a Week	10	11.1
		Thrice Daily	2	2.2
		Every 2 h	1	1.1
		As Needed	1	1.1
		Once, Not Daily	1	1.1
		Hourly Collection	1	1.1
		Total	90	100.0
4.2	Agreement on Measuring Blood Pressure at the Same Time Daily	Strongly Agree	58	63.0
		Agree	20	21.7
		Neutral	8	8.7
		Disagree	2	2.2
		Strongly Disagree	4	4.3
		Total	92	100.0
4.3	Recommended Duration for Blood Pressure Measurement	1–4 Days	3	3.3
		4–7 Days	62	67.4
		7–9 Days	27	29.3
		Total	92	100.0
5.1	Agreement on Lifestyle Changes for Hypertension Prevention & Treatment	Strongly Agree	84	91.3
		Agree	6	6.5
		Neutral	2	2.2
		Total	92	100.0
5.2	Recommended Daily Salt Intake for Hypertensives	None	23	25.6
		Half Teaspoon	41	45.5
		One Teaspoon (Correct)	23	25.6
		Two Teaspoons	3	3.3
		Total	90	100.0
5.3	Recommended Weekly Alcohol Limit for Hypertensive Men	10 Units	63	68.5
		14 Units (Correct)	27	29.3
		20 Units	2	2.2
		Total	92	100.0
5.4	Recommended Weekly Alcohol Limit for Hypertensive Women	4 Units	30	32.6
		8 Units (Correct)	54	58.7
		12 Units	8	8.7
		Total	92	100.0
5.5	Caffeine's Impact on Hypertension	1 Cup	1	1.1
		2 Cups (More than 2 Cups listed by most)	55	59.8
		4 Cups (Correct Threshold)	32	34.8
		6 Cups	3	3.3
		8 Cups	1	1.1
		Total	92	100.0
5.6	Recommended Daily Fruit and Vegetable Intake for Hypertensives	1–2 Servings	38	41.3
		3–5 Servings (Correct)	50	54.3
		6–10 Servings	4	4.3
		Total	92	100.0

### Training, continuous education, and guideline updates

Among the community pharmacists surveyed, 79.3% (N = 73) had pursued further specialised training or information concerning hypertension after obtaining their degree, indicating a commitment to further education. Nonetheless, a concerning 20.7% (N = 19) did not follow up with additional training, revealing an area for potential professional development. Updates on the most recent 2018 hypertension guidelines were afforded to only 39.1% (N = 36) of respondents, contrasting with the 60.9% (N = 56) who were advised based on outdated guidelines, highlighting a significant gap in the uptake of new scientific insights and practices (Table 3, Subtables 1.2 and 1.3).

### Patient interaction and measurement practices

Pharmacists demonstrated strong knowledge in maintaining the correct patient posture for blood pressure measurements, with 98.9% (N = 91) adhering to recommendations for seating with back support. Only one discrepancy was noted regarding the lack of back support (Table 3, Subtable 1.4). For cuff placement, a majority selected the all-inclusive correct response (71.4%, N = 65), while others picked selective components, identifying a gap that may be addressed through more extensive training on comprehensive best practices (Table 3, Subtable 1.5).

### Measurement frequency and timing

While 65.2% (N = 60) consistently adhered to measuring blood pressure twice, reflecting alignment with standard recommendations, a notable 26.1% (N = 24) opted for thrice, suggesting potential over-caution in following procedures (Table 3, Subtable 1.6). On timing intervals, the correct 1-minute gap between readings was only noted by 2.3% (N = 2), with a 5-minute interval being the most popular choice (41.6%, N = 37), indicating variability that may stem from differing training experiences or interpretations of best practices (Table 3, Subtable 1.7).

### Hypertension diagnosis and related conditions

Consensus was strong on measuring blood pressure in both arms for accurate hypertension diagnosis, with a combined 75.0% (N = 69) showing agreement (Table 3, Subtable 1.8). Pre-measurement conditions were accurately identified by 85.9% (N = 79), reaffirming the importance of adhering to preparatory guidelines (Table 3, Subtable 1.9). The usage of varied arm cuffs remains a debated topic, with 68.5% (N = 63) strongly opposing a one-size-fits-all approach, indicating awareness of individualised patient needs (Table 3, Subtable 1.10).

### Cuff size and maintenance frequency awareness

That 82.6% (N = 76) strongly acknowledged the impact of cuff size on accuracy underscores a comprehensive understanding of operational intricacies in measurement techniques (Table 3, Subtable 1.11). Yet, the frequency of sphygmomanometer maintenance showed discrepancies, with only 55.4% (N = 51) aligning with the recommended biennial schedule and others opting for varied approaches, potentially impacting device reliability over time (Table 3, Subtable 1.12).

### Understanding hypertension stages and effects

Although understanding of normal blood pressure levels was moderate (46.7%, N = 43) (Table 3, Subtable 2.1), most pharmacists correctly identified Stage 1 Hypertension levels (83.7%, N = 77), indicating solid foundational knowledge for common hypertension stages (Table 3, Subtable 2.2). However, only a third of participants correctly identified Stage 2 Hypertension levels, reflecting an area for knowledge reinforcement (Table 3, Subtable 2.3). Awareness was notably stronger for Stage 3 Hypertension, reinforcing pharmacists’ capability to recognise more severe conditions (70.7%, N = 65) (Table 3, Subtable 2.4).

### Consequences of untimely detection

Near-universal agreement (97.9%, N = 87) confirmed the understanding of potential life-threatening consequences stemming from untreated hypertension, such as stroke and cardiovascular issues, underscoring the critical role of timely diagnosis in pharmacy practice (Table 3, Subtable 3.1).

### Disease associations and screening methods

Comprehensive knowledge was further reflected in pharmacists’ ability to associate multiple diseases with increased blood pressure risks (93.4%, N = 85 choosing ‘All of the above’) (Table 3, Subtable 3.2). All respondents accurately understood the clinical nature of white coat hypertension (Subtable 3.3), and 95.7% (N = 88) affirmed pharmacy measurements as an effective screening tool for undiagnosed hypertension in communities (Table 3, Subtable 3.4).

### Home measurement and lifestyle management

Reflecting an area for improvement, only a quarter recognised the recommended home measurement frequency of twice daily (26.7%, N = 24) (Table 3, Subtable 4.1). The importance of consistent timing in measurements was widely recognised (84.7%, N = 78) (Table 3, Subtable 4.2), with acknowledgment of the appropriate measurement period at 67.4% (N = 62) (Table 3, Subtable 4.3). Lifestyle modifications were overwhelmingly supported as critical to hypertension management, yet only 25.6% (N = 23) accurately identified the appropriate daily salt intake, suggesting room for enhanced dietary counselling (Table 3, Subtables 5.1 and 5.2).

### Alcohol consumption recommendations

While the recommended weekly alcohol intake for hypertensive men was correctly identified by only 29.3% (N = 27), for women, 58.7% (N = 54) correctly identified the limit (Table 3, Subtables 5.3 and 5.4). These results suggest a need for more emphasis on nutritional education within hypertension management.

### Caffeine consumption and its impact on hypertension

The understanding of caffeine's impact on blood pressure varied among pharmacists. According to Table 3, Subtable 5.5, most believed that consuming more than two cups had an adverse effect, with 59.8% (N = 55) endorsing this view. However, the correct threshold – more than four cups – was identified by 34.8% (N = 32). This discrepancy underscores a potential area for improving knowledge about dietary triggers for hypertension, and ensuring patients receive accurate advice regarding caffeine consumption.

### Fruit and vegetable intake recommendations for hypertensives

When advising on the optimal quantity of fruits and vegetables for patients with hypertension, results showed that 54.3% (N = 50) correctly advocated for three to five servings per day (Table 3, Subtable 5.6). However, a significant proportion (41.3%, N = 38) suggested lower-than-recommended servings, indicating variability in dietary guidance provided by pharmacists.

1. Overall Knowledge Score for Hypertension Detection

The comprehensive knowledge score, assessing community pharmacists’ understanding of hypertension detection in the general population, ranged from 42.9% to 85.7%. Approximately half of the participants scored between 64.3% and 75.0%. The average knowledge score was 67.4%, with a standard deviation of ± 9.19 reflecting a moderate to high level of knowledge about hypertension detection protocols (Table 4).

2. Inferential Statistical Analysis

**Table 4.** Overall knowledge score distribution and descriptive statistics (N = 92).

Descriptive Statistics for Overall Knowledge Score	
Statistic	% Total Knowledge Score
Minimum	42.9
Maximum	85.7
Range	42.9
Mean	67.4
Standard Deviation	9.19

Frequency Distribution of Overall Knowledge Scores		
Score (%)	Frequency (n)	Percent (%)
42.9	1	1.1
46.4	1	1.1
50.0	3	3.3
53.6	7	7.6
57.1	5	5.4
60.7	9	9.8
64.3	11	12.0
67.9	14	15.2
71.4	17	18.5
75.0	10	10.9
78.6	7	7.6
82.1	6	6.5
85.7	1	1.1
Total	92	100.0

Various statistical tests were employed to examine differences in knowledge levels across different demographic and professional factors among community pharmacists. The Mann–Whitney U test was utilised for dichotomous variables, whereas the Kruskal–Wallis test was chosen for variables with more than two categories. These non-parametric tests were selected due to the ordinal nature of the data and the non-normal distribution of scores. The use of non-parametric tests allowed for robust analysis across categorical variables, providing insightful distinctions for shaping future educational and training initiatives within community pharmacy settings.

### Differences based on gender

The Mann–Whitney U test was applied to assess potential knowledge discrepancies between male and female pharmacists. The analysis considered overall knowledge scores and domain-specific areas, including blood pressure measurements in the pharmacy, hypertension categorisation, home measurement of blood pressure, and lifestyle impacts on blood pressure. The test results indicated no statistically significant differences (*p* > 0.05), suggesting that gender does not influence the knowledge level regarding hypertension management among pharmacists.

### Differences based on age

For evaluating age-related differences, the Kruskal–Wallis test was applied, given the categorical nature of age groups. Statistically significant differences were observed, highlighting younger pharmacists, particularly those aged 26–36, as having greater knowledge about lifestyle impacts on hypertension (*p* = 0.013). Additionally, overall knowledge scores were significantly lower for pharmacists over 58 years (*p* = 0.012). This indicates that younger pharmacists, possibly due to recent training or familiarity with current practices, have a heightened understanding of these aspects compared to their older counterparts.

### Differences based on highest level of education

The Mann–Whitney U test was utilised to explore differences in knowledge based on the highest level of education attained by pharmacists. The results revealed significant disparities, with pharmacists holding postgraduate degrees demonstrating superior knowledge in blood pressure measurements (*p* = 0.009) and overall knowledge scores (*p* = 0.002). This outcome underscores the value of advanced education in equipping pharmacists with the necessary skills and knowledge.

### Differences based on years of experience as a pharmacist

An analysis using the Kruskal–Wallis test revealed significant knowledge disparities related to years of experience. Pharmacists with less than 5 years of experience scored higher in understanding the impact of lifestyle on blood pressure (*p* = 0.02), and also had higher overall knowledge scores compared to those with 36–45 years of experience (*p* = 0.01). These findings suggest newer pharmacists may be more attuned to contemporary practices and emerging knowledge, reflecting a need for continuous education for more experienced pharmacists.

### Differences based on pharmacy ownership or employee status

The Mann–Whitney U test was conducted to compare pharmacists who own their pharmacies versus those employed at them. No statistically significant differences were detected (*p* > 0.05), indicating that ownership status does not impact pharmacists’ knowledge levels across the domains of hypertension management assessed.

### Differences based on the person conducting blood pressure measurements

The study utilised the Kruskal–Wallis test to determine if the person conducting blood pressure measurements (pharmacist, assistant, or patient) affected knowledge outcomes. The test found no significant differences (*p* > 0.05), which suggests that the specific individual conducting the measurements does not influence the pharmacists’ knowledge levels in managing hypertension.

### Differences based on whether post-degree training or information on hypertension guidelines was received

Using the Mann–Whitney U test, it was determined that there were no significant differences between pharmacists who received post-degree training or information and those who did not (*p* > 0.05). This indicates that additional training post-degree did not substantially affect the overall knowledge levels measured in this study.

### Differences based on whether the update was based on 2018 guidelines

The Mann–Whitney U test assessed knowledge differences based on whether pharmacists were updated following 2018 guidelines. Generally, no significant differences were noted (*p* > 0.05), except for slight significance in blood pressure measurement total scores (*p* = 0.038), suggesting minimal impact of the guideline updates on knowledge in specific areas.

## Discussion

The present study underscores the crucial role pharmacists play globally in hypertension management and screening, aligning with a growing body of international evidence supporting pharmacist-led interventions. To our knowledge, it is the first study to explore the specific knowledge, attitudes, and practices of pharmacists in the Greek context with regard to hypertension management. Hypertension is a pervasive health issue worldwide, affecting approximately 20% to 30% of adults in regions like North America (Hajjar & Kotchen, 2003), and a significant proportion of the population in other countries. Despite the well-established benefits of hypertension treatment in reducing cardiovascular risk (World Health Organization, 2023), hypertension remains under-detected and poorly controlled across various healthcare systems (Giles et al., 2009; Stergiou et al., 2021; Williams et al., 2018).

Internationally, innovative models of care are being implemented to address these challenges, with pharmacists increasingly involved in hypertension management and screening within community settings. In North America, pharmacist-led interventions have been associated with significant improvements in blood pressure control. A systematic review and meta-analysis of randomised controlled trials revealed that pharmacist interventions resulted in greater reductions in systolic blood pressure (−7.6 mmHg) and diastolic blood pressure (−3.9 mmHg) compared to usual care (Santschi et al., 2011). Such reductions are clinically meaningful, potentially reducing the risk of stroke by about 30% and myocardial infarction by 20% (Law et al., 2009).

Similarly, in Europe, studies have demonstrated the effectiveness of pharmacist-led hypertension clinics within general medical practices. One study implemented a pharmacist-led Hypertension Management Clinic in a general medical practice and found that the number of patients achieving target blood pressure levels increased from 36% pre-clinic to 85% post-clinic (Reid et al., 2005).

In Germany, community pharmacists are increasingly involved in the detection and control of hypertension, which enhances patient care. Since June 2022, several pharmacist-led services, including blood pressure control in hypertension, are reimbursed by all statutory health insurance funds and private insurance companies (Schulz et al., 2022). Structured blood pressure testing in pharmacies identified a significant number of individuals with undetected or poorly controlled hypertension. In a project involving 17 community pharmacies and 187 individuals, poorly controlled blood pressure was detected in 55% of patients with known hypertension, leading to referrals to their physicians (Schulz et al., 2020). This collaborative approach facilitates early detection and continuous management of hypertension.

In Asia, similar trends are observed. A study conducted in Indonesia revealed moderate knowledge levels among pharmacists, with a need for improved practices and attitudes towards hypertension management (Wijaya et al., 2020). This aligns with our findings in the Greek context, where pharmacists demonstrated moderate knowledge, particularly in lifestyle-related aspects of hypertension management. Conversely, a study in Pakistan reported poor knowledge levels regarding antihypertensive medications among pharmacists, highlighting variability in pharmacist knowledge across different contexts and underscores the need for tailored educational interventions (Roy et al., 2020). Additionally, this also aligns with findings in the UK, where pharmacist-led models in community settings have effectively managed hypertension (Albasri et al., 2020).

Systematic reviews and meta-analyses have further substantiated the positive impact of pharmacist-led interventions. One such review demonstrated that community pharmacist-led management of hypertension significantly changed systolic blood pressure over usual care by −6.1 mmHg (95% CI, −3.8 to −8.4 mmHg) and diastolic blood pressure by −2.5 mmHg (95% CI, −1.5 to −3.4 mmHg) (Cheema et al., 2014). These interventions often include patient education, lifestyle counselling, medication management, and regular monitoring of blood pressure.

Despite these advancements, challenges remain in fully integrating pharmacists into hypertension care pathways globally. Barriers such as time constraints, the need for specialised training and legal approvals, communication gaps between pharmacists and physicians, policy limitations, and reimbursement issues must be addressed (Jeemon et al., 2021). Addressing these barriers requires policy support, development of collaborative care models, and investment in pharmacist education and training.

The Greek context, with its unique healthcare system and cultural factors, presents specific challenges and opportunities for pharmacist education and training. The role of pharmacists in hypertension management is increasingly recognised, yet there remains a gap between potential and practice. Pharmacists’ involvement in blood pressure monitoring, patient education, and lifestyle counselling can significantly impact patient outcomes. However, to fulfil this role effectively, pharmacists must be equipped with the necessary knowledge and skills.

The study indicates that while Greek pharmacists have a reasonable understanding of hypertension management, there is room for improvement, particularly in lifestyle-related knowledge. Younger pharmacists and those with higher education levels demonstrated better knowledge. Our study suggests that enhancing the knowledge and skills of pharmacists – particularly in lifestyle-related aspects of hypertension management – is crucial. Continuous professional development and targeted training programmes focusing on lifestyle factors and patient counselling are essential to enhance pharmacists’ capabilities in hypertension detection and management. Furthermore, fostering stronger collaboration between pharmacists and other healthcare providers can lead to more integrated and effective hypertension care.

## Conclusion

In conclusion, international evidence consistently demonstrates the significant impact that pharmacists can have on hypertension management and screening within the community pharmacy setting. Leveraging their accessibility and expertise, pharmacists contribute meaningfully to improving blood pressure control, reducing cardiovascular risk, and enhancing patient outcomes worldwide. To fully realise this potential, future efforts should address implementation barriers, strengthen interprofessional collaboration, and support pharmacists in their expanding roles in hypertension care. In Greece, most community pharmacies already offer blood pressure measurement services, with pharmacists directly involved in patient assessments. However, these services are not yet integrated into a formally structured or reimbursed national programme under governmental healthcare oversight. The Panhellenic Pharmaceutical Association (PPA), alongside the Federation of the Cooperative Pharmacists of Greece (OSFE), is actively and continuously advocating for the professional recognition of pharmacists as essential healthcare providers capable of delivering advanced, structured, and officially supported health services within community pharmacy settings. While pharmacy education in Greece has traditionally focused on the pharmaceutical sciences, limited elements of pharmacy practice, clinical management, social pharmacy and patient care have gradually been integrated into the curriculum. This imbalance is partly attributed to the longstanding perception that pharmacy practice discipline/research lacks the scientific rigour of the core pharmaceutical sciences, often being characterised as a ‘soft’ science (Garcia-Cardenas et al., 2020). As a result, Greek universities continue to face a shortage of faculty members with formal specialisation in pharmacy practice and the provision of pharmacy-led advanced services.

In reality, pharmacy practice is a complex, interdisciplinary, and evidence-based field requiring advanced clinical decision-making, patient-centered care, and healthcare team collaboration. To fully prepare future pharmacists for the evolving demands of healthcare, there is an urgent need to modernise pharmacy education in Greece, aligning with the Pharmacy 2030 vision of pharmacists as providers of comprehensive healthcare services. Structured academic training and continuous professional development are essential to equip both current practitioners and pharmacy students with the knowledge and competencies required to deliver high-quality hypertension management and other advanced clinical services within the community pharmacy setting.
